# Supplementary material for: Failure to replicate the Aubert-Fleischl effect
Source: PLoS One. 2025 Dec 26;20(12):e0324420. doi: 10.1371/journal.pone.0324420 (PMC12742770; doi:10.1371/journal.pone.0324420)
Supplement: S1 Appendix — This Appendix provides additional information on a secondary analysis we conducted using (Bayesian) Generalized Linear Mixed Modelling rather than the main (Bayesian) Linear Mixed Modeling-based analysis described in the main text. (PDF) [file pone.0324420.s001.pdf]

Appendix A: Secondary Statistical Analysis using Generalized Linear Mixed Modelling

Since this project also served as a case study for a methods paper on power analyses for psychophysical designs, we also performed a secondary analysis that some of our simulations have shown to be somewhat more sensitive than the two-step approach outlined above (Jörges, 2021). This approach uses Generalized Linear Mixed Modelling (as implemented in the lme4 package for R) to fit one statistical model across all data points, rather than first fitting psychometric functions and then performing the statistical analysis over PSEs and/or JNDs (Moscatelli & Lacquaniti, 2012).

Outlier Analysis – We used the same outlier analysis related to eye-movements as before. Additionally, we excluded all data points where a sphere cloud speed was presented that was more than 3 times higher than the target speed, under the assumption that such extreme values could only occur if the participant made mistakes early in the staircase.

Statistical Analysis – To assess the impact of the Gaze condition in the No Relative Motion condition, we fitted a Generalized Linear Mixed Model with a logit link function with the participants' responses as dependent variable, Gaze Condition, the number of saccades, the speed of the sphere cloud, as well as the speed of the sphere as fixed effects, as well as random intercepts and random slopes for the number of saccades, the speed of the sphere cloud and the sphere speed per participant:

$$Response \sim Gaze\ Condition + Number\ of\ Saccades + Speed_{Cloud} + Speed_{Sphere} + (1 + Number\ of\ Saccades + Speed_{Cloud} + Speed_{Sphere} | Participant) \quad (8)$$

We fitted the 95% bootstrapped confidence intervals to assess statistical significance.

The random effect structure was determined by starting with a maximal model with random slopes for Gaze condition, number of saccades, cloud speed and sphere speed per participant. We then used Likelihood Ratio Tests (as implemented in the anova() function from base R) to determine whether this maximal model was significantly better than all simpler models. Where an additional parameter did not improve the model fit, we removed it. This led to the exclusive of random slopes for Gaze condition per participant, leaving us with the random effect structure described above.

For the Relative Motion condition, we fitted an equivalent Bayesian Generalized Linear Mixed Model:

$$Response \sim Gaze\ Condition + Number\ of\ Saccades + Speed_{Cloud} + Speed_{Sphere} + (+Number\ of\ Saccades + Speed_{Sphere} | Participant) \quad (9)$$

As for the two-step approach used in the main analysis, we used the hypothesis() function to determine the Bayes Factor associated with the Gaze condition.

In case of discrepancies between the results of the main analysis and this analysis, the main analysis prevails.
