# Supplementary material for: Failure to replicate the Aubert-Fleischl effect
Source: PLoS One. 2025 Dec 26;20(12):e0324420. doi: 10.1371/journal.pone.0324420 (PMC12742770; doi:10.1371/journal.pone.0324420)
Supplement: S2 Appendix — Appendix B provides additional graphics on important eye-behavior metrics such as gaze gains and saccades. (PDF) [file pone.0324420.s002.pdf]

Appendix B: Plots of eye behavior per participant

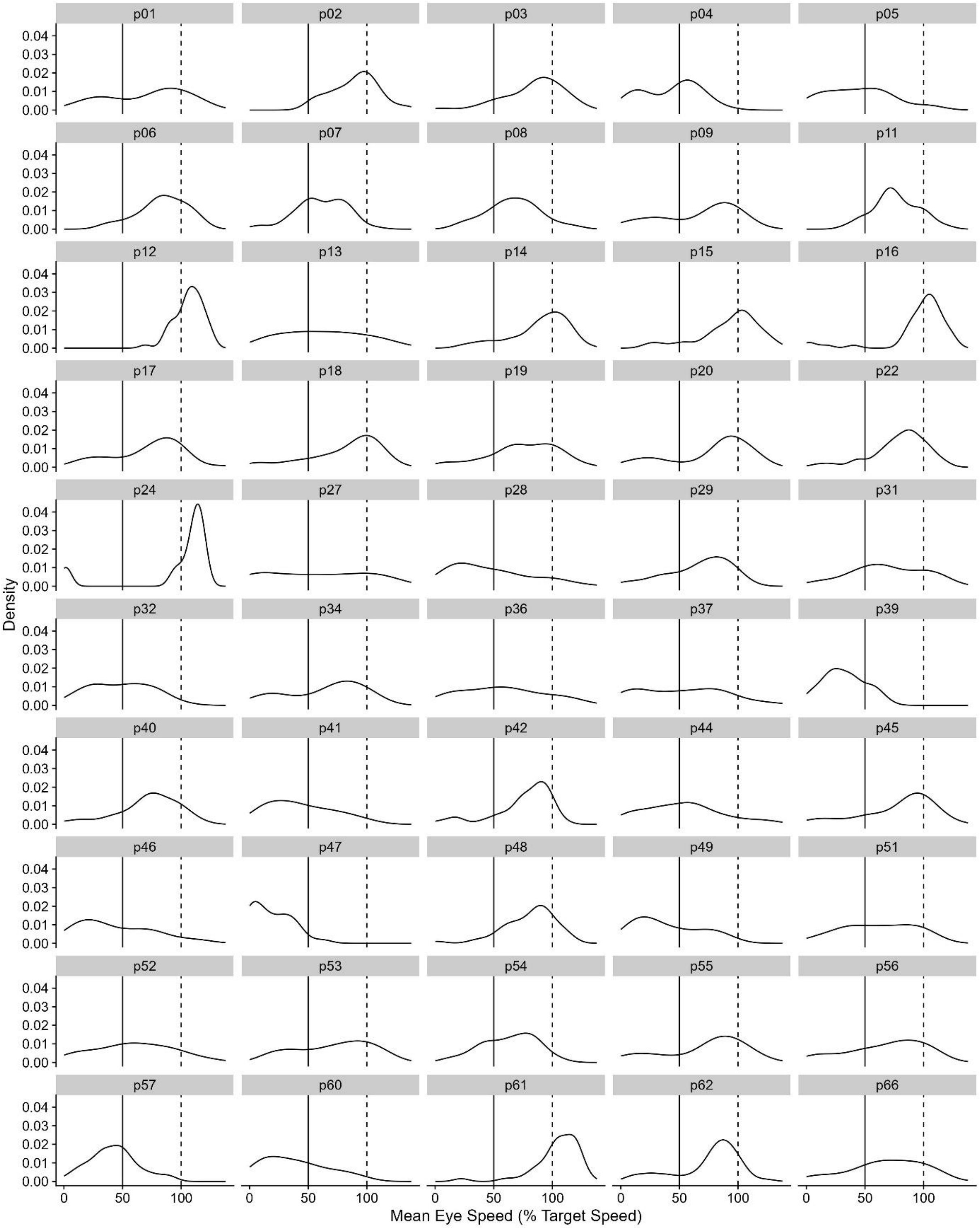

Figure A1: Distributions of mean eye speed for the sphere part of each trial for all pursuit trials per participant (panels). The solid vertical line indicates the criterion (eye speed must be at least 50% of the sphere speed), i.e., participants displayed acceptable

eye behavior for the part of the distribution to the right of the line and unacceptable ones for the part of the distribution to the left of the line. The dashed line indicates when the eyes moved at the same speed as the target (100%).

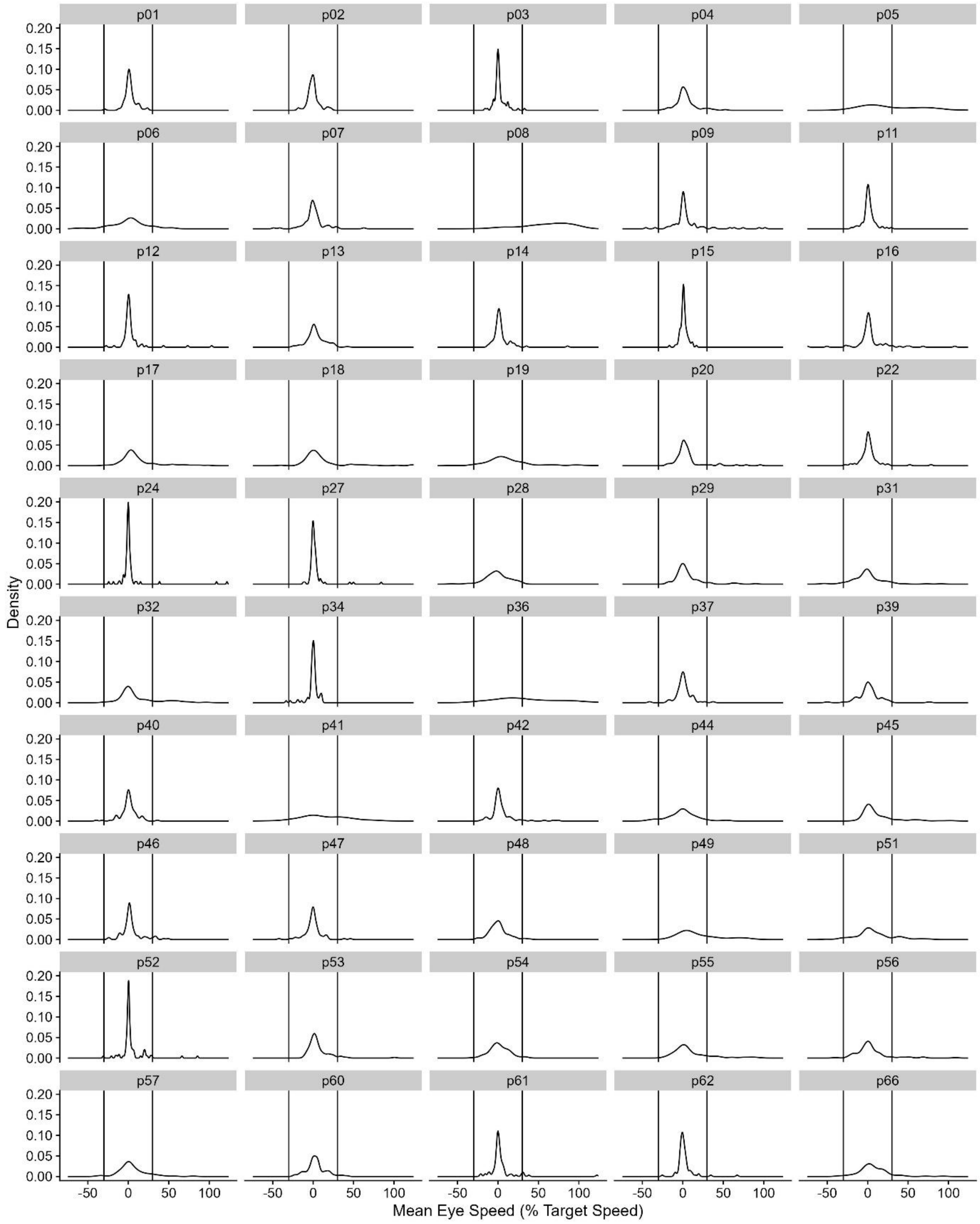

Figure A2: Distributions of mean eye speed for each trial for the sphere part of all fixation trials per participant (panels). The vertical lines indicate the criterion (eye speed must lower than 30% of the sphere speed), i.e., participants displayed acceptable eye behavior for the part of the distribution between the two vertical lines.

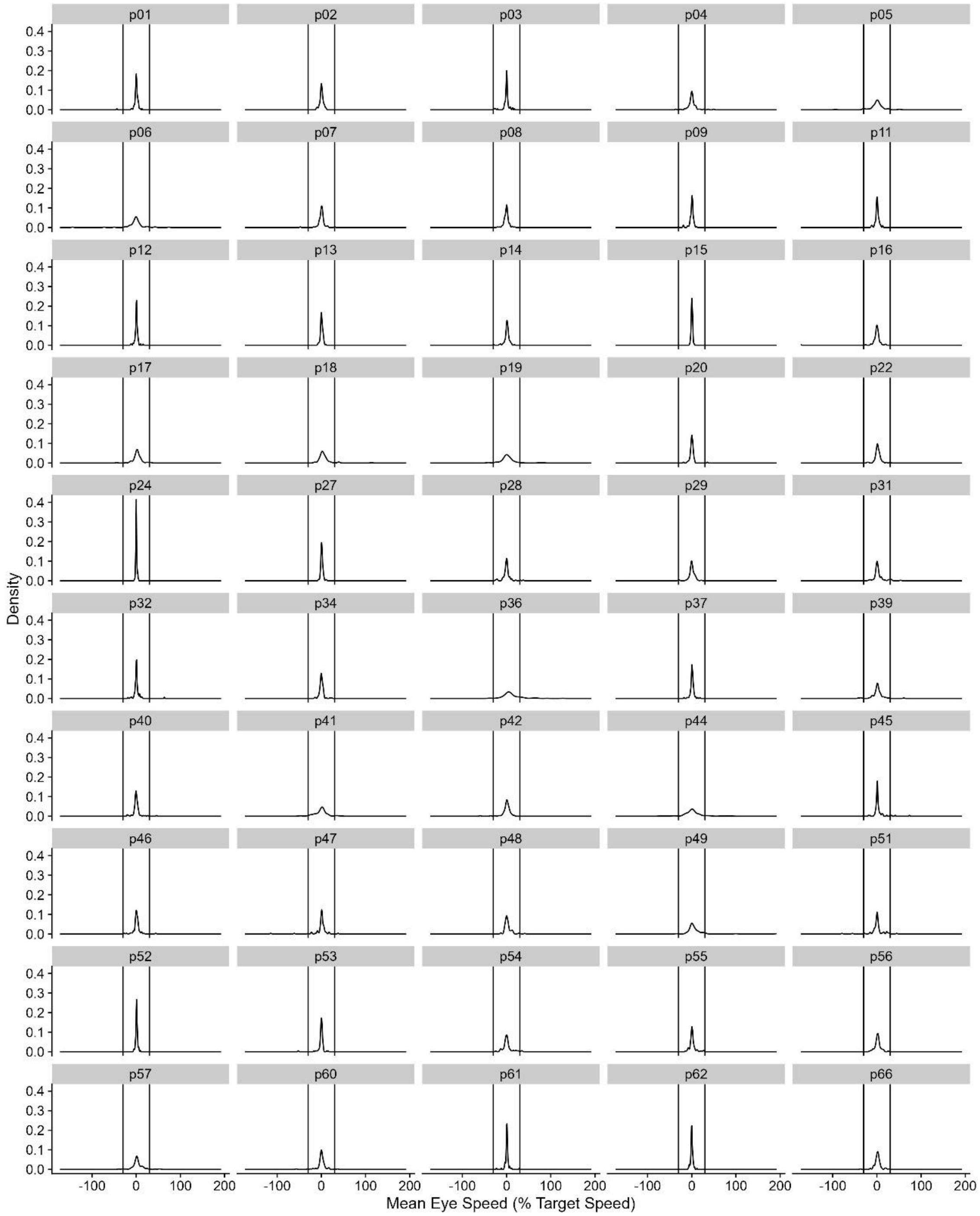

Figure A3: Distributions of mean eye speed for each trial for the sphere cloud part of all trials per participant (panels). The vertical line indicates the criterion (eye speed must be at least 50% of the sphere speed), i.e., participants displayed acceptable eye behavior for the part of the distribution between the two vertical lines.

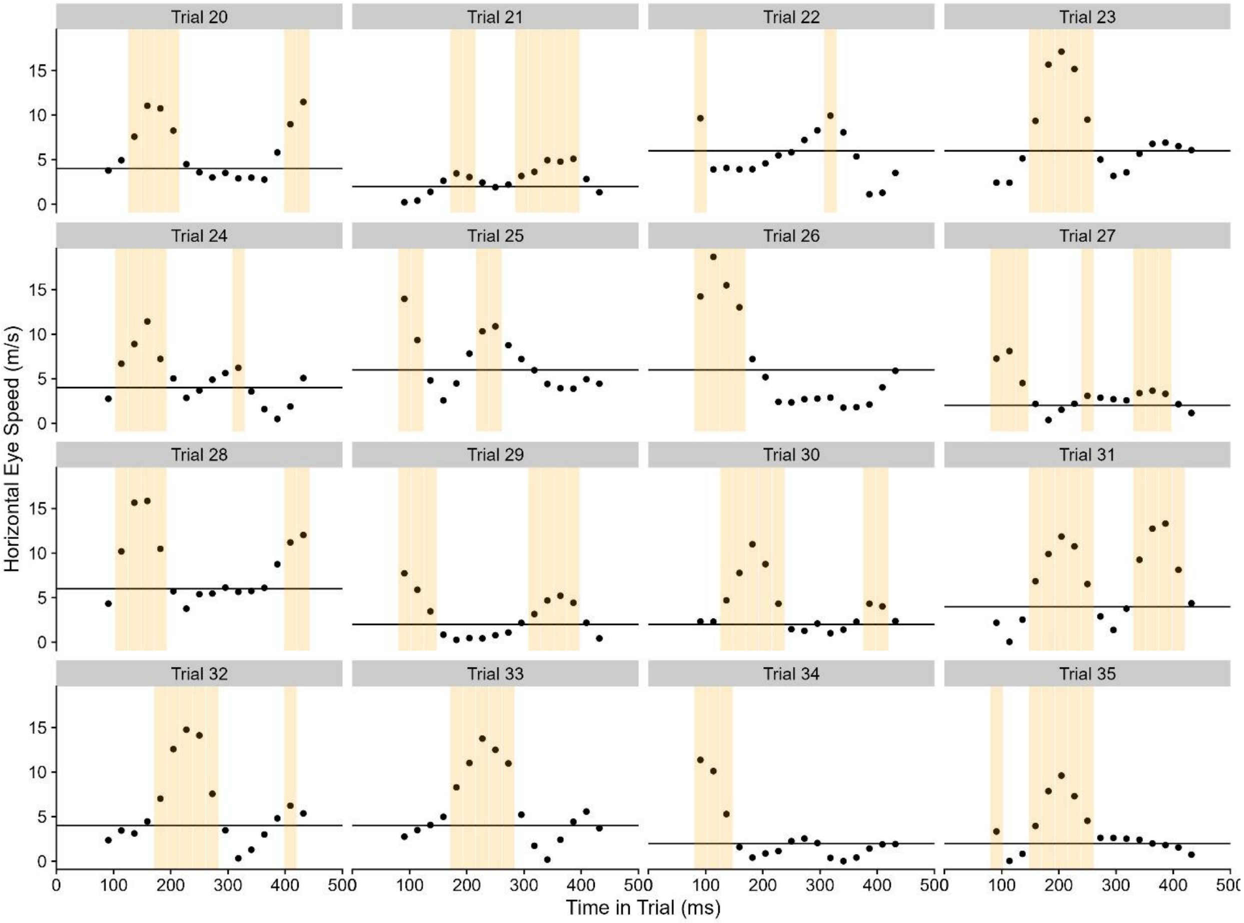

Figure A5: Horizontal eye speed (y axis) over the course of the constant speed phases (x axis) of 16 trials (panels) from a randomly drawn participant (p05). The horizontal eye speed is computed in the target plane, for ease of comparison to the target speed (horizontal line in each panel). The orange shaded areas are those frames that our algorithm flagged as saccades.

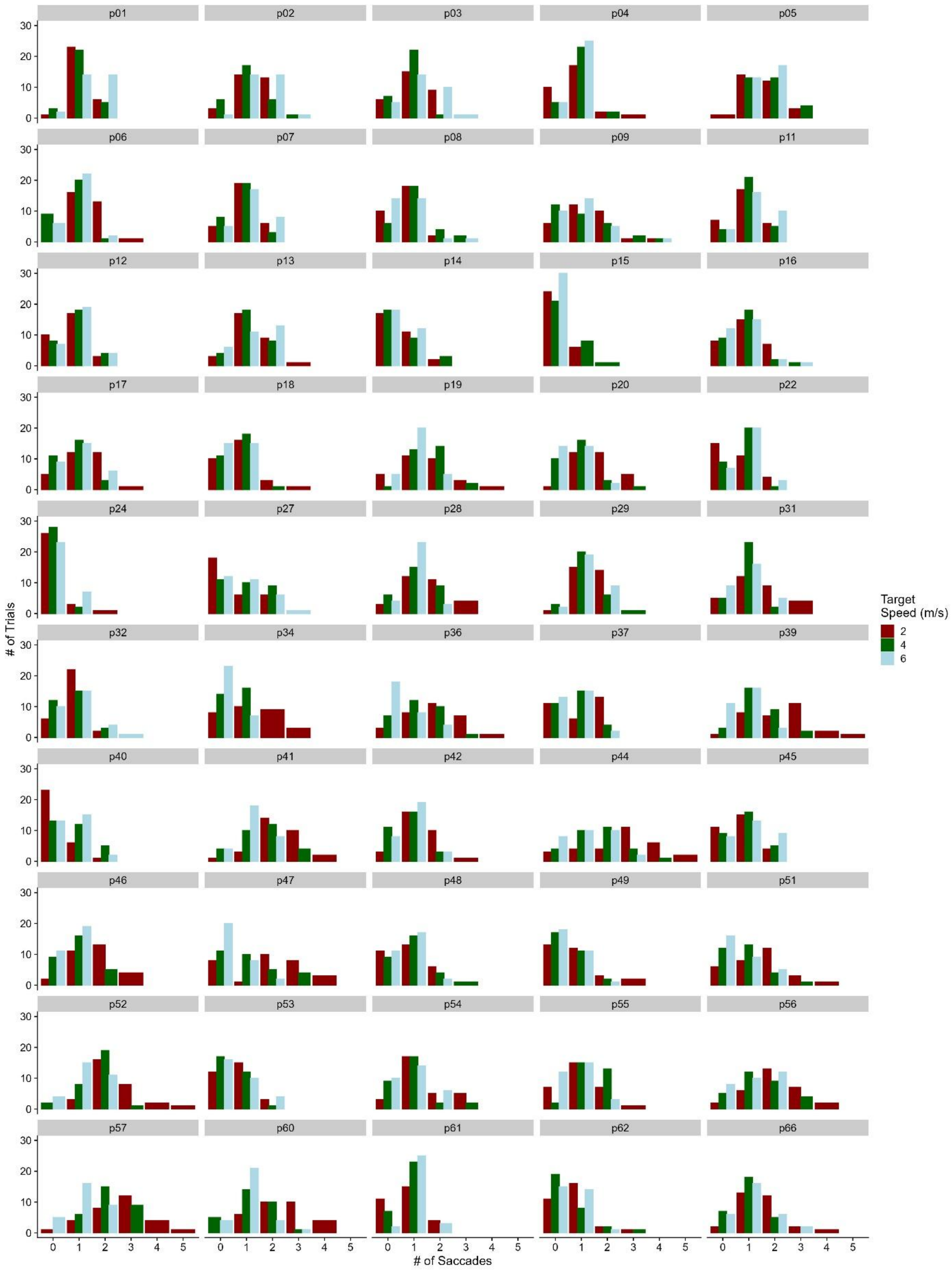

p01
p02
p03
p04
p05
p06
p07
p08
p09
p11
p12
p13
p14
p15
p16
p17
p18
p19
p20
p22
p24
p27
p28
p29
p31
p32
p34
p36
p37
p39
p40
p41
p42
p44
p45
p46
p47
p48
p49
p51
p52
p53
p54
p55
p56
p57
p60
p61
p62
p66
# of Trials
# of Saccades
Target Speed (m/s)
2
4
6

Figure A6: Number of saccades while the target was presented during the Pursuit-Relative Motion condition (y axis) divided up by target speeds (color-coded), separately for each participant (panels). This includes all trials, before outlier removal.
