# Supplementary material for: Failure to replicate the Aubert-Fleischl effect
Source: PLoS One. 2025 Dec 26;20(12):e0324420. doi: 10.1371/journal.pone.0324420 (PMC12742770; doi:10.1371/journal.pone.0324420)
Supplement: S3 Appendix — Appendix C provides a detailed breakdown of all difference contrasts relating to all variables for all statistical models reported in this paper, including 95% credible intervals and Bayes Factors where relevant. (PDF) [file pone.0324420.s003.pdf]

Appendix C – Summary of all difference contrasts of all variables in all statistical models fitted in this paper

Table A1: Regression coefficients and 95% Credible Intervals for all tested difference contrasts, as well as the Bayes Factors for all tested hypotheses.

	Difference Contrast	95% Credible Interval (Lower Bound)	95% Credible Interval (Upper Bound)	Bayes Factor (Evidence for absence of effect)
**Bayesian Linear Mixed Model (Relative Motion)**				
Intercept	0.99	0.61	1.33	
Pursuit (vs. Fixation)	0.11	-0.11	0.33	2.13
**Bayesian Linear Mixed Model (No Relative Motion)**				
Intercept	1.32	0.92	1.69	
Pursuit (vs. Fixation)	0.1	-0.17	0.36	2.21
**Bayesian Generalized Linear Mixed Model (Relative Motion)**				
Intercept	1.1	0.66	1.54	
Pursuit (vs. Fixation)	0.25	0.06	0.43	0.26
# of Saccades (increase in PSE per Saccade)	0.02	-0.05	0.09	
Ball Speed (4m/s vs. 2m/s)	0.75	0.52	0.99	
Ball Speed (6m/s vs. 2m/s)	1.88	1.57	2.2	
**Bayesian Generalized Linear Mixed Model (No Relative Motion)**				
Intercept	1.28	0.85	1.71	
Pursuit (vs. Fixation)	0.1	-0.18	0.38	5.1
Ball Speed (4m/s vs. 2m/s)	0.96	0.74	1.17	
Ball Speed (6m/s vs. 2m/s)	2.09	1.75	2.42	
**Bayesian Generalized Linear Mixed Model (Correlation)**				
Intercept	1.13	0.61	1.67	
Eye Speed (increase in PSE per m/s eye speed)	0.02	-0.03	0.06	25.19
# of Saccades (increase in PSE per Saccade)	0.02	-0.04	0.08	

Ball Speed (4m/s vs. 2m/s)	0.92	0.63	1.23	
Ball Speed (6m/s vs. 2m/s)	2.03	1.57	2.5	
**Bayesian Generalized Linear Mixed Model (Criterion)**				
Intercept	1.16	0.65	1.69	
Below Cutoff (vs. Above)	-0.05	-0.18	0.07	7.93
# of Saccades (increase in PSE per Saccade)	0.02	-0.04	0.08	
Ball Speed (4m/s vs. 2m/s)	0.96	0.68	1.24	
Ball Speed (6m/s vs. 2m/s)	2.09	1.64	2.56	
**Bayesian Generalized Linear Mixed Model (Interaction)**				
Intercept	1.07	0.65	-0.15	
Pursuit (vs. Fixation)	0.22	0.04	0.41	
No Relative Motion (vs. Relative Motion)	0.1	0.03	0.17	
Ball Speed (4m/s vs. 2m/s)	0.83	0.63	1.03	
Ball Speed (6m/s vs. 2m/s)	1.95	1.65	2.25	
Interaction (Relative Motion Condition vs. Gaze Condition)	-0.07	-0.18	0.04	7.73
**Bayesian Linear Mixed Model (Precision)**				
Intercept	3.36	2.56	4.08	
Pursuit (vs. Fixation)	0.27	-0.11	0.65	0.81
No Relative Motion (vs. Relative Motion)	-0.07	-0.46	0.32	
