# Supplementary material for: Failure to replicate the Aubert-Fleischl effect
Source: PLoS One. 2025 Dec 26;20(12):e0324420. doi: 10.1371/journal.pone.0324420 (PMC12742770; doi:10.1371/journal.pone.0324420)
Supplement: S4 Appendix — Appendix D provides additional information on gaze behavior, including detailed plots of average gains per trial as well as saccades and heatmaps of where gaze hit in the plane of the stimulus. (PDF) [file pone.0324420.s004.pdf]

Appendix D – Supplementary Analysis of Gaze Behavior

In this appendix, we provide additional information about the recorded gaze behavior. Overall, we will show here that the condition-wise eye movement characteristics are unlikely to explain the absence of the Aubert-Fleischl effect in our dataset.

Figure A7A shows how gains (after saccade removal) were distributed across the different target speeds and gaze conditions in the trials included into analysis. As a consequence of our outlier analysis, gains in the pursuit condition were at least 0.5, while gains were at most 0.33 in the fixation condition. This figure also shows that, in the pursuit Condition, gains averaged around 1, while much lower gaze gains were observed in the Fixation condition. It is also evident that ocular pursuit remained successful even for higher target speeds.

Figure A7B further supports the notion that eye behavior was executed largely as intended by showing that only one saccade was executed in a typical pursuit trial, while no saccades were more likely than two or more saccades. The fact that no saccades were executed in the vast majority of fixation trials provides further evidence that participants largely followed instructions.

This is further cemented by the fact, shown in Figure A7C, that participants kept their gaze on the fixation cross successfully while the ball sphere was on screen.

Finally, Figure A7D shows the gains in those trials that were marked as “instructions not followed” and therefore removed from our analysis.

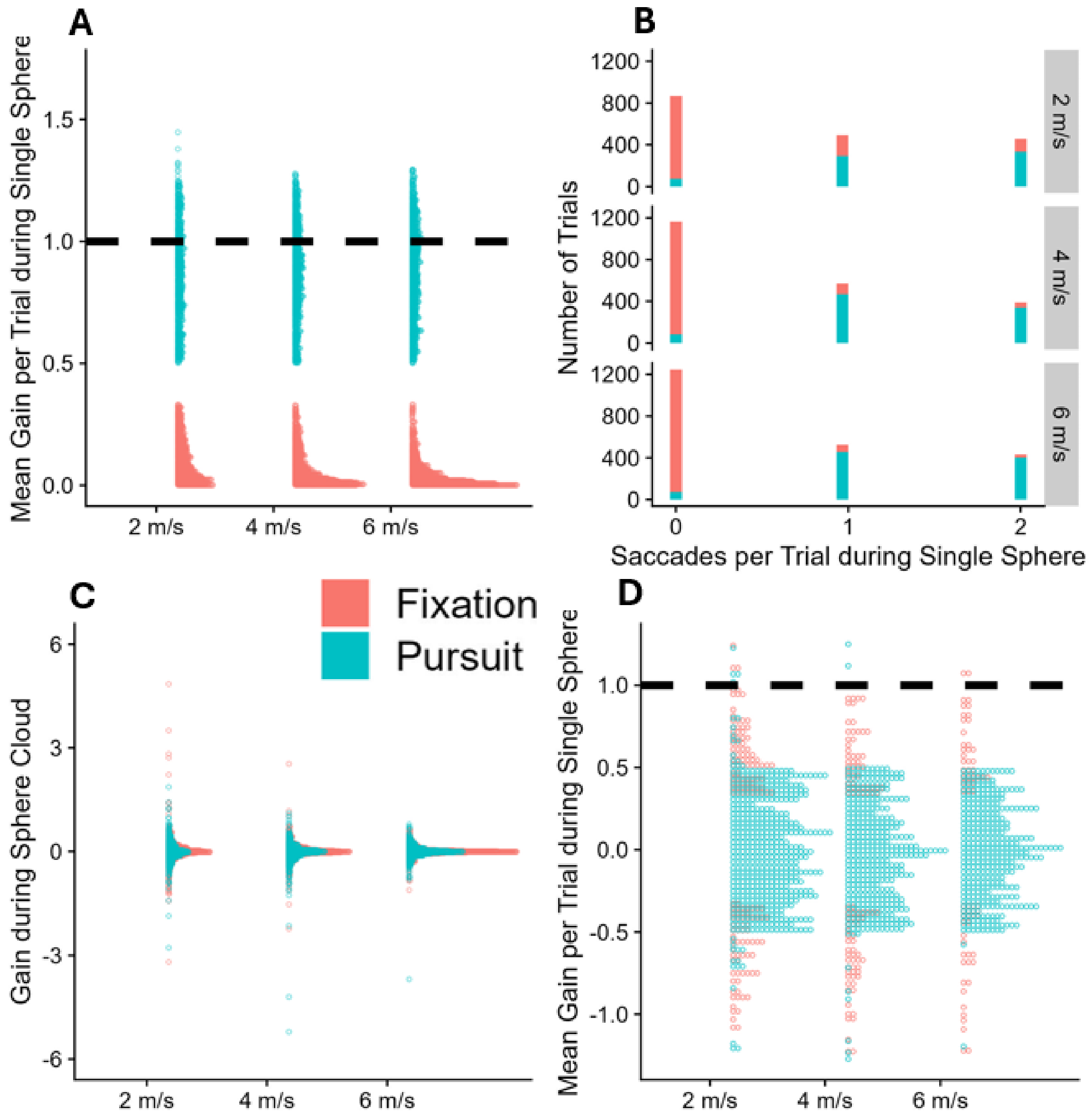

Figure A7: A. Distributions of mean gains during single sphere per trial, separately for target speeds (x axis) and gaze condition (color-coded). B. Number of trials (y axis) in which a certain number of saccades (x axis) was executed while the single sphere was on screen, separately for target speeds (horizontal panels) and gaze condition (color-coded). C. Distributions of mean gains while the sphere cloud was on display per trial, separately for target speeds (x axis) and gaze condition (color-coded). D. Distributions of mean gains while the single sphere was on display per trial, separately for target speeds (x axis) and gaze condition (color-coded). This plot shows performance for trials that were excluded during outlier removal.

Figures A8A and A8B show, respectively, the gaze positions in pursuit (A) and fixation (B) trials. The plots show that participants were largely directing their gaze at the expected locations, i.e., a band around 2.2 m for the pursuit trials (where the sphere was moving) and concentrated around the fixation cross (simulated at coordinates of [0; 1.7 m]) for the fixation condition. Since our main outlier analysis focused solely on eye speed in x direction, neglecting the height at which participants directed their gaze, we wanted to further explore to what extent this component of gaze behavior influenced speed judgements. First, we categorized all trials depending on the percentage of frames for which gaze was

directed around object height (2.2m +- 0.5m, corresponding to +- 2.5° around the 1.5° target). 16% of trials otherwise included in the analysis failed to meet this criterion in the Pursuit condition. Given this rather large share, we exploratorily repeated the main analysis after excluding these 16% of trials from the dataset. Bayes factors changed from 2.13 to 1.24 (in favor of there not being a difference between Fixation and Pursuit) for PSEs and from 0.81 to 1.62 for the JNDs. These negligible differences suggest that, as long as participants moved their eyes in the direction of the target, it did not matter whether this occurred at the height of the object or above or below.

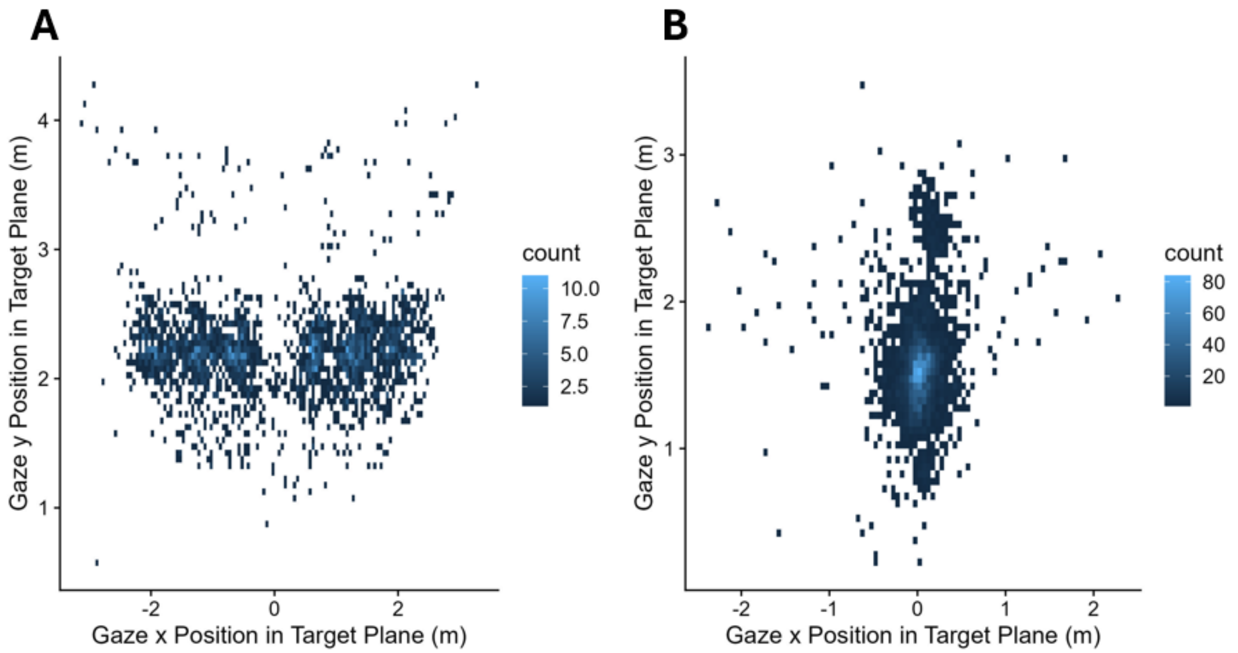

Figure A8: Heatmap of gaze positions while the single sphere was presented, for the Pursuit gaze condition (A) and the Fixation gaze condition (B). Only frames from trials included in the analysis are depicted.
