# Supplementary material for: Failure to replicate the Aubert-Fleischl effect
Source: PLoS One. 2025 Dec 26;20(12):e0324420. doi: 10.1371/journal.pone.0324420 (PMC12742770; doi:10.1371/journal.pone.0324420)
Supplement: S5 Appendix — Appendix E contains all deviations from the pre-registration (PDF) [file pone.0324420.s005.pdf]

# Deviations from the Pre-Registration

The pre-registration can be accessed here (https://osf.io/byhg8).

We made the following changes with regards to the pre-registration:

- We improved the performance of the program while the sphere cloud is on screen.
- We mistakenly described the number of trials per PEST staircase as 20 in the pre-registration. The actual number is 15. Consequently, we changed the outlier criterion regarding the maximum number of trials that were marked as "instructions not followed" to exclude conditions where 20 or more out of 30 trials did not meet the eye movement criteria, rather than 30 out of 40. Our intention was to only include those conditions where at least 10 trials satisfied the criterion.
- We added an eye-movement training in the beginning of the experiment and eye-movement tests before each experimental block.
- We changed the link function of the GLMMs from "logit" to "probit" in order to be able to use the method described by Moscatelli and Lacquaniti (2012) to obtain PSE estimates from the fitted GLMMs.
- We gave the GLMM analyses described in the Appendix of the pre-registration a more prominent role in the manuscript.
- We added three exploratory analyses that are marked as such in the manuscript.
- In response to reviewer feedback
    - we now compute the mean gaze speed per trial only based on non-saccade frames.
    - we have changed the structure of the Bayesian Generalized Linear Mixed Models.
    - we switched all remaining frequentist analyses to Bayesian analyses.
    - we added an exploratory analysis regarding the interaction between Gaze condition and Environment condition.
    - we also exclude all trials where participants executed more than three saccades.
    - We lowered the eye speed criterion for saccade identification from two times the ball speed to 1.5 times the ball speed.
- The original (pre-registered) data analysis script can be found here: https://osf.io/p9kwm
