# Supplementary material for: Failure to replicate the Aubert-Fleischl effect
Source: PLoS One. 2025 Dec 26;20(12):e0324420. doi: 10.1371/journal.pone.0324420 (PMC12742770; doi:10.1371/journal.pone.0324420)
Supplement: Attachment — Submitted filename: Response to the Reviewer PlosOne.docx [file pone.0324420.s007.docx]

Reviewer #1: This study focused on the Auber-Fleischl phenomenon, in which humans tend to perceive the speed of a moving object as slower when tracking it with their gaze than when their gaze is fixed. Previous experiments on the Auber-Fleischl effect have generally used targets moving against a blank background, but in this experiment, we hypothesized that in the case of moving objects in the real world, the relative movement of the object and the background provides additional clues, and that the visual system may compensate for the Auber-Fleischl phenomenon. Therefore, the authors conducted a two-interval forced-choice task comparing the speed of a single sphere with that of a cloud of spheres. The authors demonstrated that there was no evidence of the Aubert-Fleischl effect, specifically a reduction in the underestimation of speed during pursuit and fixation, in either environment. These results did not support the Bayesian model based on the idea that accuracy decline during tracking makes judgments vulnerable to the prior distribution of slow motion.
The hypotheses and results of this study are interesting, but there are several questions regarding whether the results obtained from the experimental methods and analysis of this study lead to the conclusions of this study, and several revisions are necessary to draw objective conclusions based on the results of this study. Specific questions are listed below.

Specific comments:

1) First, regarding the effect of eye movements on the results of this experiment, the generalized linear mixed model includes eye movement velocity as a fixed effect, and comparisons are also made between trials where eye velocity is above and below the reference value. The lack of eye movement velocity data and information on the gain of smooth pursuit or the velocity of catch-up saccades in response to stimulus velocity makes it difficult to interpret the comparison of velocity perception between fixation and pursuit conditions. For example, it is unclear whether OKR is elicited by visual motion from the stimulus during the fixation condition. It is also necessary to consider the magnitude of retinal slip based on the pursuit gain.

*Response 1: We have now added more detailed information on eye-movements in Appendix E, including comparisons of smooth pursuit speeds and number of saccades between the conditions. As can be gleaned from plot A8A (blue distribution; see below), the mean gain across all conditions is roughly 1 (0.96 for 2 m/s, 0.98 for 4 m/s, and 0.99 for 6 m/s).*

*As for the OKR, according to our outlier criterion we only included plots where the average gain across a trial was at most 0.33. The plot above shows the mean gain per trial for all included trials (red distribution). Only 9.3% of Fixation trials (387 out of 4140) were excluded from the analysis based on this criterion.*

*Further, In the vast majority of included Fixation trials (88%, or 3313 out of 3753), no saccades were executed, as is shown in the new plot A8B (red bars).*

*Please also note that trial with more than 3 saccades were excluded from the analysis.*

2) Among the target movement speeds used in this study, it seems possible to maintain a certain degree of pursuit gain at 15 deg/s, but at speeds of 30 and 40 deg/s, it is difficult to maintain high gain, so the gain is likely to be low and many catch-up saccades are likely to be mixed in. In such cases, it is easy to imagine that significant retinal slip will occur and that the frequency of catch-up saccades will have a significant impact on perception. In other words, when comparing fixed and pursuit conditions at different stimulus speeds, it is necessary to consider the accuracy of eye movements. Furthermore, many previous studies that demonstrated the overt Fleischl effect used relatively slow stimulus speeds within the range where pursuit gain could be maintained (e.g., less than 15 deg/s). Therefore, when comparing the results of this study, which used relatively fast conditions, with previous studies, it is necessary to consider pursuit gain. When making such comparisons, unless the dynamics of eye movements are clearly demonstrated, it is difficult for readers to understand the factors that led to these results.

*Response 2: We have now included plots with condition-wise comparisons of gain and number of saccades per trial in Appendix E (plot A8). These plots show that, perhaps surprisingly, eye behavior is fairly uniform across stimulus speeds. As per our outlier analysis, we also don’t include any trials where the gain was below 0.5; and the average gain was around 1 (0.96 for 2 m/s, 0.98 for 4 m/s, and 0.99 for 6 m/s), as expected.*

3) In addition, when the background is textured, if the subject can ignore the texture information of the background to a certain extent, the pursuit gain is maintained, but if attention is directed to the background, the gain is expected to decrease further. Therefore, it is necessary to objectively evaluate the eye movements of the subjects in comparison with the JND shown in this result.

*Response 3: As a crude proxy to whether participants were paying attention to the stimulus or to the background, we divided the dataset (after our initial outlier analysis) into trials where the gaze landed within a 1m vertical band around the actual stimulus height (+/- 2.5° visual angle around the 1.5° target) in 80% or more of the frames versus such trial where this criterion was not satisfied. We then re-analyzed the data after excluding trials that did not satisfy the criterion (14.9% of the data) and found that the results did not change meaningfully (Bayes Factors for the absence of an effect of gaze condition of 2.9 (outliers not removed) and 2.7 (outliers removed)). Similarly, results only changed marginally for the JNDs, from 1.5 to 1.8.*

*We have added the following heatmaps of the x-y distribution of the gaze data in Appendix D (A. for the pursuit condition and B. for the fixation condition):*

4) The results of this study indicate that the perceived speed in the pursuit condition was numerically higher than that in the fixation condition in both the relative motion condition and the no relative motion condition. This shows the opposite effect to that reported in the previous study by Aubert-Fleischl, but it is probably the result of significant retinal slip and many catch-up saccades occurring in the pursuit condition.

*Response 4: We have now added summary plots of gains (A8A, C and D) and number of saccades (A8B) for all experimental conditions in Appendix D. Figure A8B shows that the most likely number of saccades per trial was one and it was more likely to observe no catch-up saccade than two or more saccades, potentially thanks to the pursuit entrainment period in our stimulus. It is therefore unlikely that the absence of a positive result is due to retinal slip or a large number of catch-up saccades.*

[4-cont] In particular, in the tracking condition, information from eye movement reference copies, proprioception, and retinal slip is combined to estimate motion. However, each of these information sources contains inherent noise, and one possible cause of unstable velocity estimation during integration is the presence of this noise. Can the inherent noise be estimated from the eye movement data in this experiment?

*Response 5: Given that our experiment was not designed to disentangle noise from different sources, we can’t tell which of these sources was dominant in our design. However, JNDs did not differ between Fixation and Pursuit, indicating that noise introduced by target tracking did not influence the overall noise level meaningfully.*

*This may be because other sources of noise were much more influential, or because any precision penalties incurred by tracking were offset by gains in sensitivity during tracking, as reported by de la Malla et al. (2022).*

*Reference:*

*Malla, C. de la, Smeets, J. B. J., & Brenner, E. (2022). Pursuing a target with one’s eyes helps judge its velocity. Perception, 51(12), 919–922.* [*https://doi.org/10.1177/03010066221133324*](https://doi.org/10.1177/03010066221133324)

5) Regarding the moderate evidence that there was no difference in PSE between trials that met the eye movement criteria and those that did not, what eye movement dynamics were exhibited in trials that did not meet the criteria? For example, why is the eye velocity so slow in cases that do not meet the criteria? Without this information, it is difficult for readers to interpret the results.

*Response 6: Since trials where the eye velocity did not meet the criteria occurred in both fixation and pursuit trials, and for all target speeds, it is hard to draw causal conclusions as to why participants failed to follow the target with their gaze based on the data.*

*Speculatively, we would consider factors such as inattention and lack of motivation to play major roles here: the experiment took more than an hour to complete, and our participants were undergraduates who had no extrinsic motivators to achieve a certain level of performance as their course credit was guaranteed just by virtue of them showing up for the study.*

*We have included a summary plot of the eye speeds in the rejected trials in the appendix for further reference (Figure A8D).*

Reviewer #2: The basic idea of the manuscript is reasonable and the experiment seems to be competently done. Still, I am puzzled by many aspects of the manuscript. Maybe a revision could make a difference; I am not sure.

Things that concern me include:

1. I don't understand what the sphere cloud looks like. I tried to watch the video links to OSF, but the videos don't play. (As I type of my review, I realized that if I download the mpg files, I can play them locally, but they don't play in my web browser. That helps.) I do think the text should better explain the stimuli.

*Response 7: We have added detail about the sphere cloud stimulus in the manuscript.*

2. The pre-registration document is not much of a pre-registration. It feels more like a draft of the manuscript that was written after gathering pilot data. It is similar to a pre-registration document, but not quite. The outlier criteria different from what is in the pre-registration document, without comment or justification.

*Response 8: We have been writing our preregistrations in the style of Stage 1 Registered Reports; i.e., we write the first half of the manuscript (introduction, methods, and we often also include a results section on the pilot data we have collected) and publish it as preregistration. We then collect the data and add the final Results and Discussion sections to the preregistration document (in the style of a Stage 2 Registered Report). We are happy to receive feedback on which information the reviewer is missing from our preregistration in order to add it in the future.*

*We have further verified our preregistered and final outlier analyses and couldn’t find any (unreported) changes beyond a typo where we wrote 30% in the manuscript when the preregistration said 33% (which was also implemented in our analysis code).*

3. When removing the texture wall, the authors used black, which then had the impact of preventing measure of eye movements. Why not just use a non-textured (e.g., white) wall?

*Response 9: We wanted to eliminate relative motion between the target and any possible reference. When using a white background in a VR headset, participants might have been able to use the edges of the headset screen as reference. When using a black background (along with the filters we attached to the screens as described in the manuscript) fully eliminated the edges’ visibility.*

4. Many of the results and conclusions seem similar to those of Dichgans et al. (1975). Why did this current study need to be run?

*Response 10: Our study aimed to approach the Aubert-Fleischl effect in more naturalistic situations like, for example, human interaction with discrete targets. While Dichgans’ single edge is in some ways similar to our sphere stimulus, there are important aspects that separate the two studies:*

- *the retinal trace drawn by a spherical object does not fit neatly into the spatial frequency-based narrative espoused by Dichgans et al. A single moving box could be interpreted as two moving edges, but the retinal pattern elicited by a sphere are much more complicated. And given that many moving objects humans interact with in real life are spherical, this warrants additional scrutiny.*
- *Interactions between background and real-life objects may therefore also be different than for a single edge moving across a screen.*
- *We are using a separation (in depth) between target and background, while the Dichgans et al. study does not separate target and background spatially.*

*We would further like to point out that Dichgans et al. only studied a sample size of 10 participants (per experiment). Therefore, even though we would largely predict our results based on these authors’ results, our much larger sample size (n = 50) is an important contribution in and of itself.*

*Finally, there are also studies in the literature that contradict the Dichgans et al. conclusions, where the Aubert-Fleischl effect was found for single targets (e.g., the original Aubert and Fleischl studies where the target was a single glowing wire in a dark room.) It was therefore useful to add further evidence to this controversy using this type of stimulus.*

*We have included this information in the manuscript.*

5. I find it annoying that the multiple links to the OSF are to different projects. Why not put everything together in one project? (Just to be clear, I very much appreciate the authors making everything available, but it confusing to distribute things across different projects.)

*Response 11: All links in the manuscript lead to the same project, but for the reader’s convenience we included links to the individual items we were talking about (e.g., the stimulus videos, the program used for stimulus presentation, the data, the code for analysis). The Open Science section further includes a link to the overall repository. We have made this clearer in the Open Science section of the manuscript.*

6. The power analysis is weird, and possibly unnecessary. The authors correctly note that trying to do a Bayesian "power analysis" is computationally challenging, but that seems like little motivation to just do a frequentist power analysis. It possibly unnecessary because a Bayesian analysis doesn't even really have a concept of Type I or II error. You could have just added participants until you got strong enough result (one way or another).

*Response 12: Initially, part of our analysis was going to be conducted in a frequentist framework (see preregistration). In response to feedback from an earlier submission, we switched the planned frequentist analyses to Bayesian analyses as well. We have therefore removed the power analysis from the Appendix and amended the remainder of the manuscript accordingly.*

7. The manuscript has quite a few minor grammar issues. I marked up the ones I noticed (along with some additional comments/corrections) in the attached PDF.

*Response 13: Thank you, this is highly appreciated.*

***We would also like to respond to one of the comments Reviewer 2 shared with us as comments in the manuscript:***

P10: The reviewer notes that, conceptually, our hypothesis should be tested using an interaction term.

*Response 14: We specifically expected the absence of any difference between Pursuit and Fixation. An interaction, on the other hand, could be found when there was a small effect in opposite directions. While it certainly is possible to capture these specific predictions with an interaction analysis, it seemed therefore cleaner to split the analyses.*
