# Supplementary material for: Failure to replicate the Aubert-Fleischl effect
Source: PLoS One. 2025 Dec 26;20(12):e0324420. doi: 10.1371/journal.pone.0324420 (PMC12742770; doi:10.1371/journal.pone.0324420)
Supplement: Attachment — Submitted filename: Revisions PLoS ONE #2.docx [file pone.0324420.s009.docx]

6. Review Comments to the Author

Reviewer #1: The authors have responded to my previous comment by presenting additional data and figures. However, since this study was unable to accurately record eye velocity during tracking, it is still questionable whether the study's conclusions can be drawn for the following reasons.

First, regarding smooth pursuit gain, it is difficult to determine whether the eye movement data presented in these results is accurate. For example, researchers who have accurately recorded human smooth pursuit understand that tracking speeds above 30 deg/s with a gain of 0.98 or higher are nearly impossible. Furthermore, it is a well-known fact that even if the eye velocity momentarily reaches the stimulus velocity during pursuit of a constant-speed stimulus, maintaining a steady velocity is difficult. For example, the following paper (Behling S, Lisberger SG. J Neurophysiol. 2023, 130(3):652-670, Figure 2) presents eye velocity trajectories for constant-speed stimuli. Maintaining velocity becomes even more difficult for high-speed stimuli. As demonstrated by the authors in this study, it is objectively difficult to believe that pursuit can be tracked with a gain of 0.98 or higher for stimuli of 30 deg/s and 45 deg/s. Furthermore, Figure A7A presents results where pursuit gain significantly exceeds 1.0, but it is generally considered nearly impossible for pursuit velocity to overshoot stimulus velocity. In addition, while overshoot may occur during the initial phase of pursuit, it does not persist continuously. This means that to clarify the velocity perception obtained in the study, one must carefully examine the effects of eye movement and retinal slip velocity; otherwise, one encounters a barrier preventing understanding of the essence.
Therefore, it is essential to first prove whether the smooth pursuit gain demonstrated in this study is objectively correct when compared to prior research. Alternatively, if this proves difficult, it should be presented as a limitation of this study. Conclusions should then be drawn under the premise that the velocity perception obtained in this study may be influenced by eye movements and retinal slip velocity.

*Response #1: One reason our gains might be higher than expected is that, due to our criterion, perhaps some frames of recorded eye movements that are already part of a saccade are still classified (see Figure 5A). We have therefore changed our analysis pipeline to use a more liberal saccade criterion (eye speed > 1.5 * target speed rather than eye speed > 2 * target speed). With this new criterion, mean gains across a trial are now much more in line with the expected gains reported in the literature; we find mean gains of 0.86 (for the 2 m/s stimulus), 0.87 (4 m/s) and 0.88 (6 m/s).*

*When using this new criterion, results of our main statistical analyses are very similar to what we found previously – if anything there is now slightly more support for PSEs being higher in the Pursuit condition than in the Fixation condition (i.e., an inverted Aubert-Fleischl effect).*

Reviewer #2: The revised manuscript is much improved. In particular, small changes to the text make it easier to understand the stimuli and task. Still, there are a few issues.

1) There are still quite a few grammatical issues. Some of these are carried over from my previous review. I, again, marked up a copy of the manuscript with some corrections and comments.

*Response 2: Our sincerest apologies. We have now made sure to address all grammatical issues in the current document.*

2) The pre-registration document at the OSF is a bit problematic. There are some deviations in the manuscript that are not explained. Worse, the OSF site indicates that the pre-registration document was modified September 25, 2025, which means it was modified since the submission of the original manuscript. If so, then it is no longer a pre-registration document. As a result, I think it is wrong for the manuscript to refer to analyses being preregistered. Maybe there were, but readers cannot verify the situation with the online materials.

*Response: Out intention was to only include those conditions where instructions were followed properly for at least 10 trials. With that, the deviation pointed out by the reviewer was implicitly covered by the deviations bullet point “We mistakenly described the number of trials per PEST staircase as 20 in the pre-registration. The actual number is 15.” However, we failed to elaborate how this related to the outlier criterion. We have now made this clear in the Open Science section of the manuscript.*

*We have also contacted OSF regarding the “Date modified” of our preregistration document. Misleadingly, “Date modified” refers to the last date anything in the repository was changed. The version history of the preregistration file can be accessed through the “Revisions” tab on the upper right (*[*https://osf.io/xcaq3/files/byhg8*](https://osf.io/xcaq3/files/byhg8)*) and shows that the preregistration has been untouched since before data collection started.*
